# Supplementary material for: Conformational Stability of Poly (N-Isopropylacrylamide) Anchored on the Surface of Gold Nanoparticles
Source: Materials (Basel). 2021 Jan 18;14(2):443. doi: 10.3390/ma14020443 (PMC7831095; doi:10.3390/ma14020443)
Supplement: Supplementary file 1 [file materials-14-00443-s001.pdf]

materials

MDPI

Article

Conformational Stability of Poly (N-Isopropylacrylamide) Anchored on the Surface of Gold Nanoparticles

Runmei Li [1], Cong Cheng [1], Zhuorui Wang [1], Xuefan Gu [2], Caixia Zhang [1], Chen Wang [1] and Xinyue Liang [3] and Daodao Hu [1,*]

1 Engineering Research Center of Historical and Cultural Heritage Protection, Ministry of Education, School of Materials Science and Engineering, Shaanxi Normal University, Xi'an 710062, China; lrm@snnu.edu.cn (R.L.); congcheng2017@snnu.edu.cn (C.C.); wangzhuorui@snnu.edu.cn (Z.W.); zcx@snnu.edu.cn (C.Z.); ccw1128@snnu.edu.cn (C.W.)

2 College of Chemistry and Chemical Engineering, Xi'an Shiyou University, Xi'an 710065, China; XuefanGu@xsyu.edu.cn

3 Institute of Industrial Hygiene of Ordnance Industry, Xi'an 710065, China; ancybz@163.com

* Correspondence: daodaohu@snnu.edu.cn

Citation: Li, R.; Cheng, C; Wang, Z.; Gu, X.; Zhang, C.; Wang, C.; Liang, X.; Hu, D. Conformational Stability of Poly (N-Isopropylacrylamide) Anchored on the Surface of Gold Nanoparticles. *Materials* **2021**, *14*, 443. https://doi.org/10.3390/ma14020443

Received: 13 December 2020
Accepted: 14 January 2021
Published: 18 January 2021

Publisher's Note: MDPI stays neutral with regard to jurisdictional claims in published maps and institutional affiliations.

Copyright: © 2021 by the authors. Submitted for possible open access publication under the terms and conditions of the Creative Commons Attribution (CC BY) license (http://creativecommons.org/licenses/by/4.0/).

The IR spectrum of prepared PNIPAM-SH is shown in Figure S1A, the peaks at 3288, 2975, 2873, 2929, 1638,1541 cm^{-1} are ascribed to NH stretching, $-CH_3$ asymmetric stretching, $-CH_2$ asymmetric stretching of carbon bone for PNIPAM, $-CH_3$ symmetric stretching, C=O stretching (amide I) and N-H stretching (amide II), respectively [1]. It also displays the $-CH_3$ deformation at 1386 and 1365 cm^{-1} [1]. ^1H NMR spectroscopy of PNIPAM-SH confirms the existence of amide and all peak assignations are shown in Figure S1B. All these characteristic peaks are perfectly correspond to the structure of. The molecular weight was calculated using a polystyrene (PS) calibration. Figure S1C displays the GPC spectrum for prepared PNIPAM-SH. It shows an unimodal peak, suggesting the absence of any disulphide coupling products. The number-average molecular weight (Mn) and the molecular weight distribution (Mw/Mn), which were determined by GPC, are 7786 g/mol and 1.095, respectively. Figure S1D gives the synthetic route of SH-PNIPAM. Briefly, it used the RAFT method, CPD as a ARFT agent, ACPA as an initiator and NIPAM as monomer. The first step obtained a pink intermediate product, and then treated by TCEP and 2-ethanolamine to obtain white target product SH-PNIPAM.

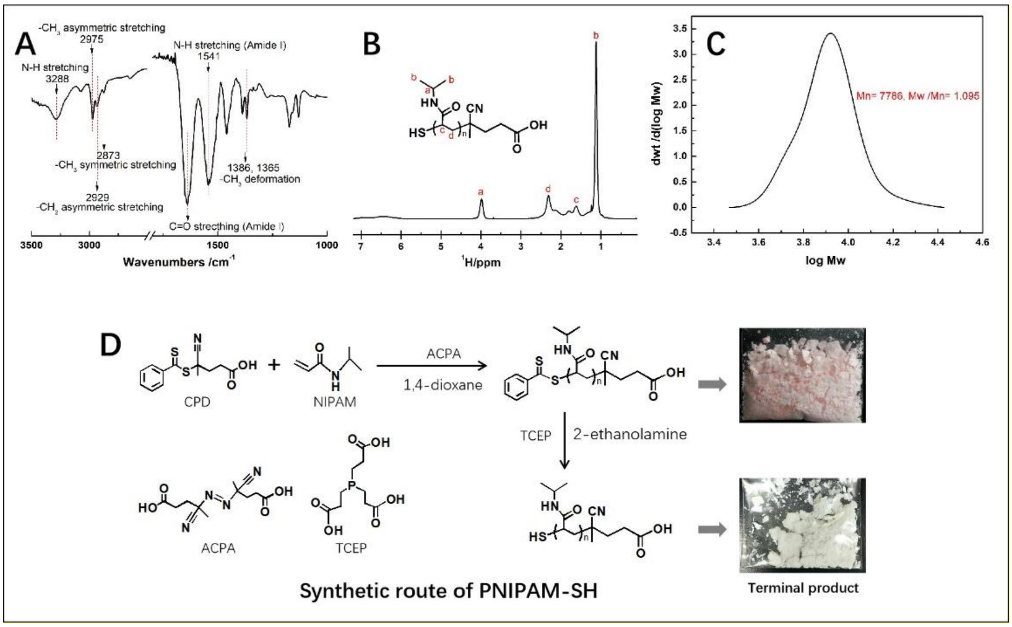

Figure S1. The spectra of IR (**A**), ¹H NMR (**B**), GPC (**C**) and synthetic route (**D**) for the prepared PNIPAM-SH.

From Figure S2A, for AuNPs, from room temperature to 60 °C, the intensity of the broad peak located at 200–300 nm gradually decreases and down to the minimum at 70 °C. Besides, an obvious shoulder peak at 220 nm appears, especially the temperature was up to 90 °C. The above phenomenon maybe related to the change of citrate. According to the reported literature, the spectral region around 200–300 nm is related to gold interband transitions and depends directly on the gold atoms concentration in the sample [3]. For AuNPs, this peak decreasing with temperature might relate to the precipitation of aggregated AuNPs in the solution. (corresponding to the decrease in SPR peak of AuNPs in Figure 2B). In this study, AuNPs solution was obtained by centrifuging to remove excess citrate, which leads to a decreased charge density on the surface of AuNPs. At the same time, increasing temperature promotes the desorption of citrate [4]. Theses two effects make Zeta potential distinctly decreased, resulting in the aggregation of AuNPs.

When the temperature exceeds 70 °C, the broad peak in the range of 200–300 nm basically disappears. Instead, a shoulder peak appears in the range of 200−230 nm, especially at 90 °C. The study indicated that this spectral change is related to the characterized absorbance of citrate [5]. When the temperature increasing, according to the Boltzmann distribution, the probability of reaching higher vibrational levels within the electronic ground state increases with increasing temperature [6]. Thus, the shoulder peak in the range of 200–230 nm gradually enhances at 70–90 °C. From Figure S2B, when the AuNPs were loaded with PNIPAM, the spectral absorbance in 200–300nm barely has change below 60 °C. It because the PNIPAM shell inhibit the aggregation of AuNPs-PNIPAM. And the explanation for the situation when the temperature higher than 60 °C is similar to that for AuNPs above-mentioned. The above contrast results further indicates the tethered PNIPAM improve the thermo-stability of AuNPs. Figure S2C,D are the UV-Vis spectra for AuNPs and AuNPs-PNIPAM solution in presence of salt changing with temperature. The profile of the spectra are similar to their no-salt counterparts but the intensity distinctly decrease compared with Figure S2A,B which are absence of salt. This reduced intensity mainly attribute to AuNPs aggregation caused by the decreased Zeta potential absolute value induced by salt effect whether for AuNPs or AuNPs-PNIPAM. The aggregation causes a lower concentration of colloid, and thus results in a relatively weak absorbance of citrate.

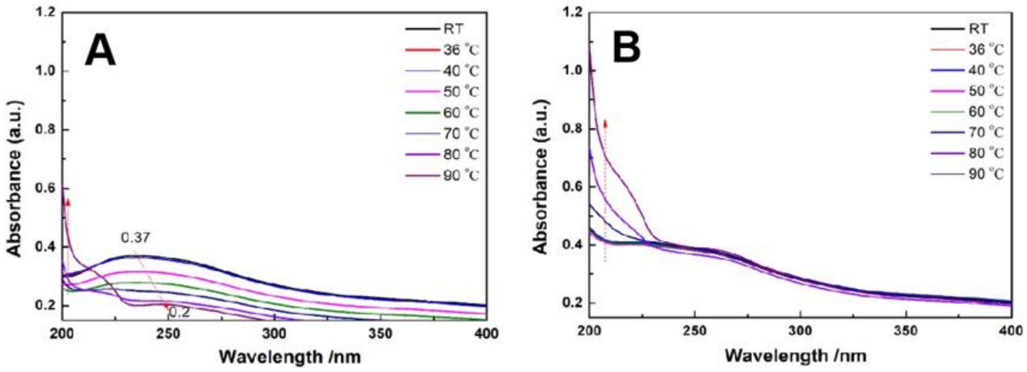

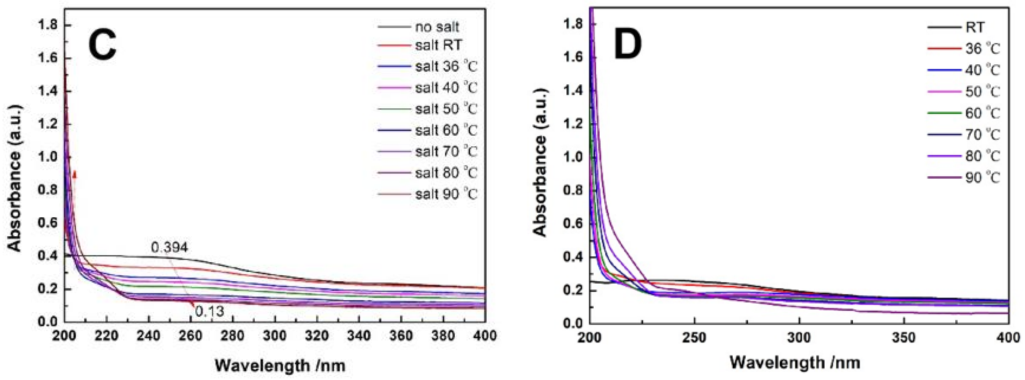

Figure S2. The change of UV spectra with temperature for AuNPs (**A**) absence of NaCl; (**C**) presence of 16.9 mM of NaCl and AuNPs-PNIPAM (**B**) absence of NaCl; (**D**) presence of 16.9 mM of NaCl.

Generally, the conformational change of PNIPAM leads to the markedly transformation between the hydrophilicity and hydrophobicity. If this transformation occures for the AuNPs-PNIPAM system, the aggregation of AuNPs-PNIPAM can appears. Unavoidably, there is a sharp change in the peak intensity and wavelength of SPR spectrum with temperature for the AuNPs-PNIPAM. Figure S3 indicates that the peak intensity of SPR spectrum for AuNPs-PNIPAM under higher salt concentration is almost linearly related to temperature rather than nonlinear, indicating that there is not the evident conformational change of PNIPAM.

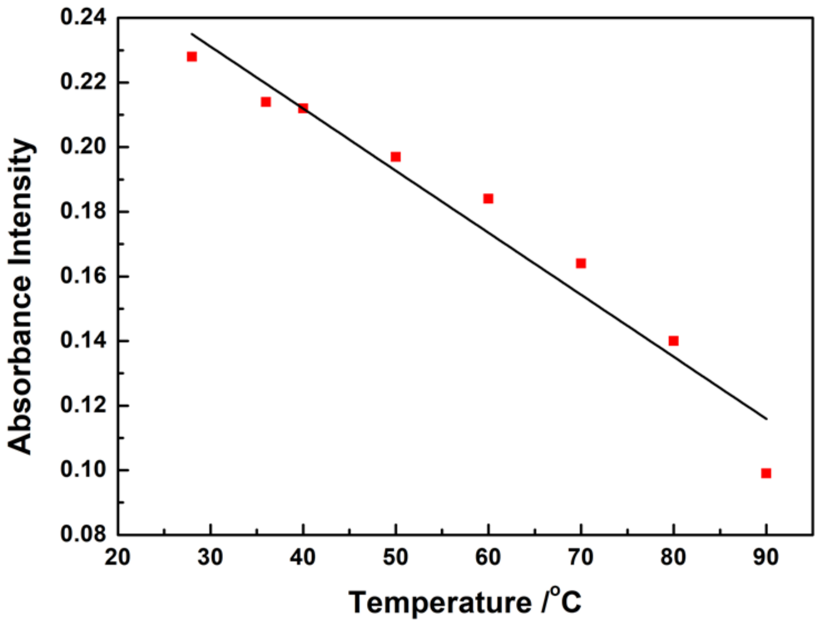

Figure S3. The relationship between the peak intensity of SPR spectrum and temperature for AuNPs-PNIPAM under higher salt concentration (16.9 mM).

In order to get the effect of diffusion of proton on the the Zeta potential of AuNPs-PNIPAM, Figure S4 shows the change in the Zeta potential for AuNPs-PNIPAM after addition of HCl (0.002M) with time. The Zeta potential values of Au-PNIPAM are −28.1 eV, −4.6, −3.6, and +5.15 eV, respectively, at 0, 2.5, 4, and 7 hours after introducing HCl, indicating that protons are slow to diffuse into PNIPAM palisades and change the Zeta potential from negative to positive one for AuNPs-PNIPAM.

For the UV-Vis spectrum of AuNPs-PNIPAm, there is the slow change in the maximum absorbance and the red-shift of maximum absorption wavelength with time after addition of HCl. Differently, for the UV-Vis spectrum of AuNPs as shown in Figure S4, after adding HCl for 1 minute, two peaks appear including typical SPR peak and the characteristic peak ranging from 600 nm to 900 nm regarding aggregation. The remarkable difference between AuNPs and AuNPs-PNIPAM, suggesting that the interaction among PNIPAM molecules tethered on the AuNPs surface is strong to inhibit the diffusion of protons.

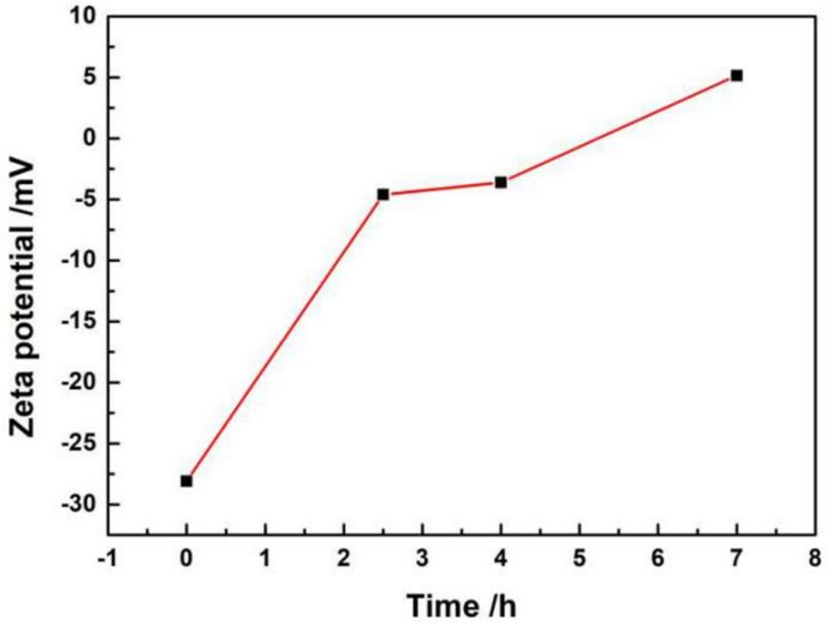

Figure S4. The change in the Zeta potential for AuNPs-PNIPAM after addition of HCl (0.002M) with time.

As the temperature above the LCST, if the change in the conformation of the immobilized PNIPAM molecules occurs, the alkyl chains of CTAB would interact with the hydrophobic segments located at the outer edge of PNIPAM palisades. Inevitably, the adsorbed positively charged CTAB molecules would reduce the net charge of AuNPs-PNIPAM, thus triggering the aggregation of AuNPs-PNIPAM. However, Figure S5 shows the UV-Vis spectrum varying with temperature for AuNPs-PNIPAM solution containing CTAB. The spectrum does not response to temperature change, revealing the failure of the conformation change of anchored PNIPAM in this situation. The experimental fact indicates that the immobilized PNIPAM on AuNPs does not have the function of thermo-sensitive conformational transformation similar to that of free PNIPAM.

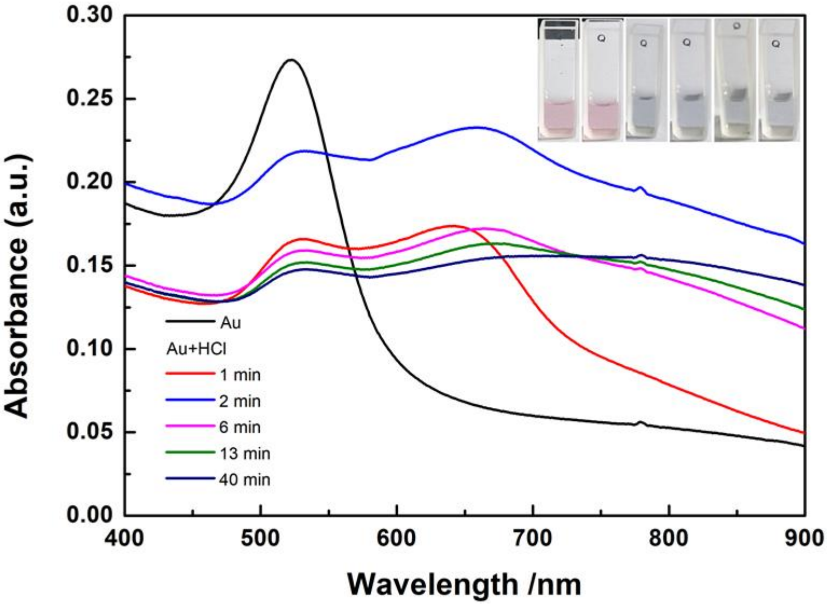

Figure S5. The change in the UV-Vis spectra with time for AuNPs after addition of HCl (0.002 M).

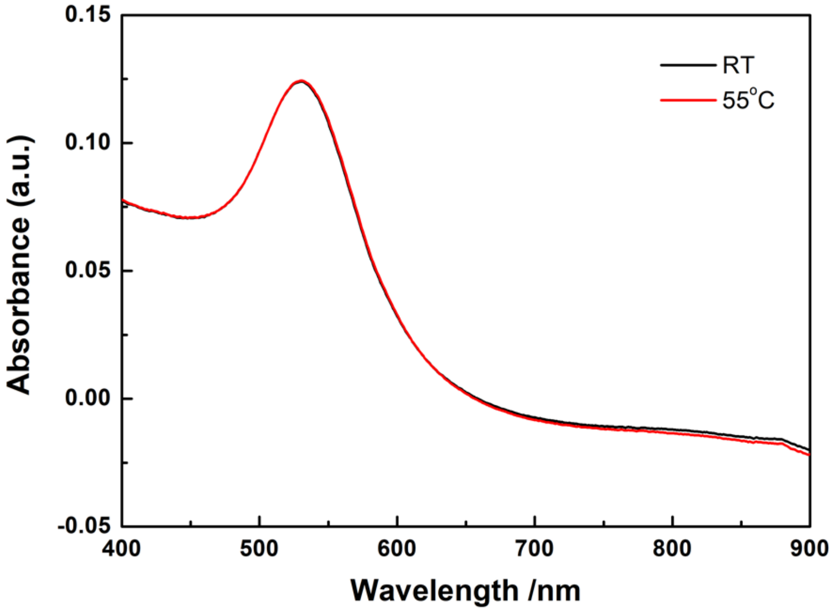

Figure S6. The UV-Vis spectra for AuNPs-PNIPAM in presence of CTAB at RT and 55 °C.

References

1. Pan, Y.V.; Wesley, R.A.; Luginbuhl, R.; Denice D.D.; Ratner, B.D. Plasma Polymerized N-Isopropylacrylamide: Synthesis and Characterization of a Smart Thermally Responsive Coating. *Biomacromolecules* **2001**, *2*, 32–36.
2. Ke, X.X.; Wang, L.; Xu, J.T.; Du, B.Y.; Tu, Y.F.; Fan, Z.Q. Effect of local chain deformability on the temperature-induced morphological transitions of polystyrene-b-poly(N-isopropylacrylamide) micelles in aqueous solution. *Soft Matter* **2014**, *10*, 5201–5211.
3. Amendola, V.; Meneghetti, M. Size Evaluation of Gold Nanoparticles by UV-vis Spectroscopy. *J. Phys. Chem. C* **2009**, *113*, 4277–4285.
4. Dutta, A.; Paul, A.; Chattopadhyay, A. The effect of temperature on the aggregation kinetics of partially bare gold nanoparticles. *RSC Adv.* **2016**, *6*, 82138–82149.
5. Krukowski, S.; Karasiewicz, M.; Kolodziejski, W. Convenient UV-spectrophotometric determination of citrates in aqueous solutions with applications in the pharmaceutical analysis of oral electrolyte formulations. *J. Food Drug Anal.* **2017**, *25*, 717–722.
6. Retzer, U.; Ulrich, H.; Bauer, F.J.; Will, S.; Zigan, Lars. UV absorption cross sections of vaporized 1-methylnaphthalene at elevated temperatures. *Appl. Phys. B* **2020**, *126*, 1–6.
